# A framework for assessing local transmission risk of imported malaria cases

**DOI:** 10.1186/s40249-019-0552-6

**Published:** 2019-06-07

**Authors:** Lei Lei, Jack S. Richards, Zhi-Hong Li, Yan-Feng Gong, Shao-Zai Zhang, Ning Xiao

**Affiliations:** 1 Jiangxi Provincial Center for Disease Control and Prevention, Nanchang, China; 2 The Macfarlane Burnet Institute for Medical Research and Public Health Ltd, Melbourne, Australia; 3 Department of Medicine, University of Melbourne, Melbourne, Australia; 4 National Institute of Parasitic Diseases, Chinese Center for Disease Control and Prevention; Chinese Center for Tropical Diseases Research; WHO Collaborating Centre for Tropical Diseases; National Center for International Research on Tropical Diseases, Ministry of Science and Technology; Key Laboratory of Parasite and Vector Biology, Ministry of Health, Shanghai, China

**Keywords:** Imported malaria, Transmission risk assessment, Analytic hierarchy process, Delphi method

## Abstract

**Background:**

A key issue in achieving and sustaining malaria elimination is the need to prevent local transmission arising from imported cases of malaria. The likelihood of this occurring depends on a range of local factors, and these can be used to allocate resources to contain transmission. Therefore, a risk assessment and management strategy is required to identify risk indexes for malaria transmission when imported cases occur. These risks also need to be quantified and combined to give a weighted risk index score. This can then be used to allocate the resources to each administrative region to prevent transmission according to the degree of risk.

**Methods:**

A list of potential risk indexes were generated from a literature review, expert consultation and panel discussion. These were initially classified into 4 first-level indexes including infection source, transmitting conditions, population vulnerability and control capacity. Each of these was then expanded into more detailed second-level indexes. The Delphi method was then used to obtain expert opinion to review and revise these risk indexes over two consecutive rounds to quantify agreement among experts as to their level of importance. Risk indexes were included in the final Transmission Risk Framework if they achieved a weighted importance score ≥ 4. The Analytic Hierarchy Process was then used to calculate the weight allocated to each of the final risk indexes. This was then used to create an assessment framework that can be used to evaluate local transmission risk in different areas.

**Results:**

Two rounds of Delphi consultation were conducted. Twenty-three experts were used at each round with 100% recovery rate of participant questionnaires. The coordination coefficients (*W*) for the two rounds of Delphi consultation were 0.341 and 0.423, respectively (*P* < 0.05). Three first-level indexes and 13 second-level indexes were identified. The Analytic Hierarchy Process was performed to calculate the weight of the indexes. For the first-level indexes, infection source, transmitting conditions, and control capacity, the index weight was 0.5396, 0.2970 and 0.1634 respectively. For the three top second-level indexes, number of imported malaria cases, *Anopheles* species, and awareness of timely medical visit of patient, the index weight was 0.3382, 0.2475, and 0.1509 respectively.

**Conclusions:**

An indexed system of transmission risk assessment for imported malaria was established using the Delphi method and the Analytic Hierarchy Process. This was assessed to be an objective and practical tool for assessing transmission risk from imported cases of malaria into China.

**Electronic supplementary material:**

The online version of this article (10.1186/s40249-019-0552-6) contains supplementary material, which is available to authorized users.

## Multilingual abstracts

Please see Additional file 1 for translations of the abstract into the five official working languages of the United Nations.

## Background

Malaria is caused by *Plasmodium* parasite infections and is transmitted by female *Anopheles* mosquitoes. The World Health Organization (WHO) and many other international agencies have set a formal public health policy for regional malaria elimination and eventual global eradiation [1]. China is well advanced in achieving this objective and has had no indigenous cases reported since 2017 [2]. Shanghai and Jiangxi Province have not had any indigenous cases for greater than 3 years, and have therefore successfully achieved sub-national malaria elimination. Imported malaria cases however, pose a significant threat to this elimination agenda. There were 2861 malaria cases in China in 2017, 2858 of which were imported while 3 were infected due to transfusion. To consolidate recent achievements and prevent the re-establishment of malaria imported into China, it is necessary to establish a practical tool to assess transmission risk when imported cases occur. Some studies were conducted for assessment or prediction of malaria transmission and variables such as population movement, number of case, temperatures, rainfall, humidity, *Anopheles*, and interventions were applied. A limited number of studies focus on refine these variables through the combination with Delphi method and Analytic Hierarchy Process (AHP). The Delphi method is widely recognized as a scientifically rigorous and practical prediction method, which can make full use of expert knowledge, experience and wisdom to achieve the goal of group decision making [3, 4]. The AHP is a comprehensive statistical method, which has a wide field of application. It quantifies the importance or priority of each factor from a systematic perspective [5, 6]. The combination of these two methods can be used to select the main risk indexes and provide a quantitative risk assessment to guide decision-making [7, 8]. With Delphi method and AHP, the purpose of this study was to develop an index system that can be used to assess local transmission risk when cases of imported malaria occur.

## Methods

### Development of evaluation indexes

**Fig. 1:**
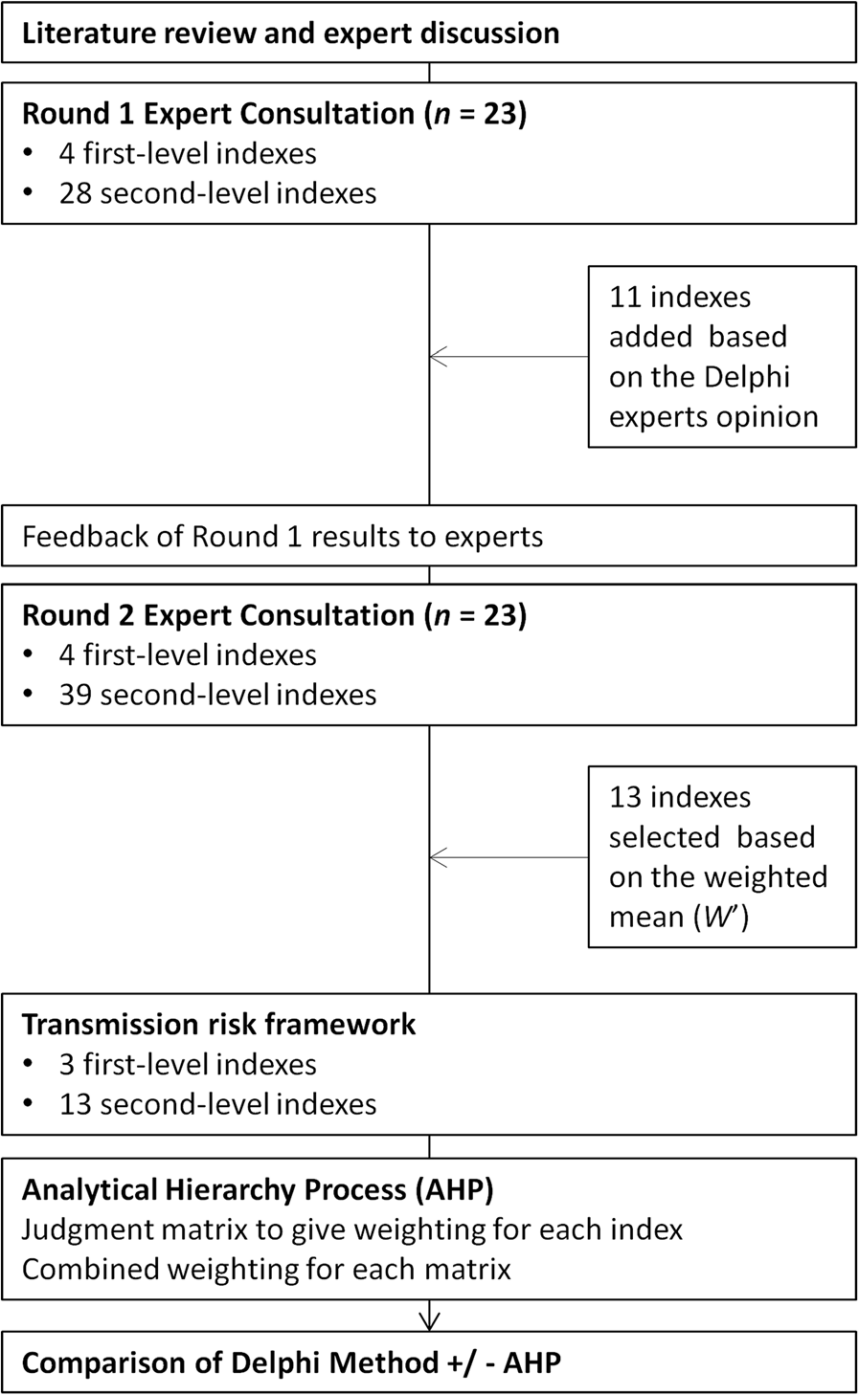
The flowchart of study process

In order to construct a preliminary index system for the Delphi questionnaire, we undertook a literature review. We searched Published articles from databases of PubMed, Web of Science, and China National Knowledge Infrastructure with the keywords of ‘imported malaria’ in conjunction with ‘risk assessment’. We also reviewed the World Malaria Report published by the WHO and Malaria Control Manual published by the China National Health Commission, both of which include indexes that impact on malaria transmission. Expert consultations were conducted and a final panel discussion undertaken with professionals specializing in malaria prevention and control to create two levels of risk index. The first-level indexes comprised of four general risk types that included: infection source, transmitting conditions, population vulnerability and control capacity. The second-level indexes were expanded, detailed aspects of each of these first-level indexes. After this process, a preliminary Delphi questionnaire was developed and a pilot survey undertaken. After this evaluation, a framework for the transmission risk assessment was formed. The flowchart of study process is shown (Fig. 1).

### Selection of Delphi experts

Twenty-three experts were selected for the Delphi consultations. The selection criteria are the followings: the ability for participants to understand the content of the questionnaire and the implications of the related indexes, professional level and number of years of experience, gender (five of 23 were female) and the geographical level of their work responsibilities (e.g. national-level, provincial-level, prefecture-level and county-level). All experts worked in centres for disease control and prevention or institutions of parasitic disease prevention and control, and were predominantly involved in malaria control and prevention, infectious diseases control or vector control. The characteristics of the experts are summarized in Additional file 2.

### Delphi consultation process

Two rounds of Delphi consultations were conducted. The questionnaire was sent to the experts by E-mail after receiving their prior approval. Experts rated each of the first-level and second-level indexes according to their level of perceived importance; rated between 1 and 5 (Additional file 3). Experts then defined the basis of their judgment according to whether their view was based on theoretical knowledge, practical experience, learned from peers or intuitive feeling; thus giving a judgment criteria coefficient between 0.1 and 0.5 (Additional file 3). Experts then rated their degree of familiarity with each of the indexes between 0 and 1 (Additional file 3). The results of each consultation round were assessed for completeness and experts were contacted if there were any omissions or if any clarifications were required. The results of the first round consultation were analysed and provided to experts when the second-round questionnaire was distributed.

### Inclusion and exclusion of the indexes

When reviewing the results of this first round of consultations, it was possible to include new indexes on the basis of direct outcomes of the consultation process, but it was not possible to remove indexes. After the second consultation round, the inclusion criteria of the results were the weighted mean of the importance score ≥ 4 (i.e. weighted mean from all the experts). At the same time, the likely implications, significance and data availability of each evaluation index were fully considered to screen and construct the assessment index system.

### Calculation of the index weight and construction of the judgment matrix

The Analytical Hierarchy Process (AHP) was then used to define the weight of each index. According to the constructed assessment index system, a judgment matrix of paired comparisons was created. This was used to define the relative significance of each index, for each hierarchy of indexes. Scales of 1–9 were used to compare the relative importance of the same hierarchy. Finally, the initial weight of each level and the combination weight of each index were calculated.

### Statistical analysis

#### Calculation of expert’s authority coefficient, the importance score

The Expert authority coefficient was calculated as follows: Cr = (Ca + Cs) /2 (Ca referring to the expert’s judgment criteria for the indexes, and Cs referring to the familiarity degree for the indexes).

The importance score was represented by a weighted mean $W^{\prime }=\sum \limits_1^{\mathrm{n}}\mathrm{Crn}\ast \mathrm{Sn}/\mathrm{n}$ (n referring to the number of the expert and S referring to the importance coefficient of the index). The Kendall’s coefficient of concordance *W* statistic is a non-parametric statistical method that used to calculate the expert coordination coefficient *W*.

#### Calculation of the weight of each index in AHP

The initial weight coefficient and normalized weight coefficient of each index were calculated as follows:

Initial weight coefficient ${\mathrm{w}}_i^{\prime }=\sqrt[m]{a_{i1}\ast {a}_{i2}\ast \cdots {a}_{im.}}$

Normalized weight coefficient ${\mathrm{w}}_i={{\mathrm{w}}_i}^{\prime }/\sum \limits_{i=1}^m{{\mathrm{w}}_i}^{\prime }$

(*i* refers to row, *m* refers to column, *a* refers to paired comparison value).

#### Consistency test

In order to test whether the calculated weight coefficient conforms to logic, we calculated the consistency index (CI). The formula was as follows:

Consistency index $$ CI=\frac{\lambda_{\mathrm{max}}-m}{m-1} $$

Maximum characteristic root ${\lambda}_{\mathrm{max}}=\sum \limits_{i=1}^m{\lambda}_i/m$, ${\lambda}_i=\sum \limits_{j=1}^m{a}_{ij}{W}_j/{W}_i$

In order to measure the satisfactory consistency of different judgment matrix, the average random consistency index (CR) was calculated as follows:

Average random consistency index $CR=\frac{CI}{RI}$

The values of *RI* are shown in Table 1. If CI < 0.1, it could be considered that the relative priority order of indexes was not logically confused. If CR < 0.1, the judgment matrix could be considered to have satisfactory consistency.

**Table 1:** Value of 1–9 hierarchy average random consistency index *RI*

Hierarchy	1	2	3	4	5	6	7	8	9
*RI*	0	0	0.58	0.9	1.12	1.24	1.32	1.41	1.45

The Excel 2010 software was used to establish the database. All the questionnaire data were double entered. Data analysis was performed using Microsoft Excel 2010 (Microsoft Corporation, Redmond, Washington, USA) and SPSS 20.0 software (IBM Corporation, Armonk, New York, USA).

## Results

### Development of a framework for evaluation indexes

Through a literature study, expert consultations and a panel discussion, an initial transmission risk assessment framework was established that included four first-level and 28 secondary-level indexes (Additional file 4). First-level indexes included infection source, transmitting conditions, population vulnerability, and control capacity. The second-level indexes were expanded concepts derived from each of these first-level indexes. After analysis and feedback from the first round of Delphi consultation, 11 additional second-level indexes were added to give extra detail about the travel history, liquidity of people in incubation period, data of large livestock (pigs, cows, etc.), varieties and quantities of insecticide, *Anopheles* transmission energy (blood-sucking habit, biting rate, etc.), drug resistance, awareness among medical staff, diagnostic capacity, blood test capacity, highly sensitive screening tools, standardized treatment. The second round consultation assessed these four first-level and 39 s-level indexes (Additional file 4).

### Delphi expert consultation

#### Basic information of the experts

A total of 23 experts participated in the consultation, among whom 78.26% were males (18/23) and 21.74% were females (5/23). The experts ranged in age from 39 to 60 years, with an average age of 48.9. The number of years working in parasite control ranged from six to 42 years, with an average length of 22.5 years. With respect to the academic level of the experts, 60.87% (14/23) were professors, and 39.13% (9/23) were associate professors. Five experts were national-level, while 12 were provincial-level from eight provinces including Yunnan, Anhui, Henan, Jiangsu, Hubei, Zhejiang, Hebei and Jiangxi, and six were prefecture and county-level. Their major areas of expertise included malaria prevention and control, infectious disease control, vector control and other fields. In both rounds, 23 questionnaires were issued and recovered with the recovery rates of 100%.

#### Expert’s authority coefficient

According to the expert self-evaluation scores, the authoritative coefficients for the 23 experts on various indicators all reached more than 0.80. The mean first round authority coefficient was 0.90 (minimum 0.84, maximum 0.96), while it was 0.90 (minimum 0.80, maximum 0.97) in the second round. Generally, it was considered that an expert’s authority coefficient greater than or equal to 0.70 was in an acceptable range [9]. It showed that the experts in the survey had a high level of familiarity with the indexes, including research and practical work in these areas. Therefore, the selection of indexes and the results had high credibility.

#### Degree of expert coordination

Kendall coordination coefficient (*W*) refers to whether there are big differences between experts in their opinions on an evaluation of each index. *W* is between 0 and 1, with a greater value, indicating a higher degree of concordance between experts. In the first round consultation the experts’ coordination coefficient (*W*) = 0.341, *P* < 0.05 (*χ*^2^ = 243.413, *P* = 0.000), and in the second round consultation the experts’ coordination coefficient (*W*) = 0.423, *P* < 0.05(*χ*^2^ = 409.050, *P* = 0.000). This indicated that the concordance of expert opinion was good. Compared with the results of the first round, the coordination degree of the second round was higher.

#### Establishment of the index framework

A transmission risk assessment index framework was built after the second round of Delphi consultation. This comprised of 3 first-level and 13 secondary-level indexes (Table 2). Only indexes with an importance score (Weighted mean) ≥ 4 were included in the final framework.

**Table 2:** Transmission risk assessment index system

Index type	Assessment index	Weighted mean(W')
First-level index	A Infection source	4.7500
	B Transmitting conditions	4.5978
	C Control capacity	4.3739
Second-level index	A1 No. of imported cases	4.5391
	A2 Types of imported cases	4.3500
	A3 Awareness of timely medical visit of patient	4.4217
	B1 Anopheles species	4.5804
	B2 Anopheles density	4.3565
	C1 Prevention and control system	4.4087
	C2 Financial support	4.1217
	C3 Staff training	4.0065
	C4 Work execution	4.3870
	C5 Availability of drugs	4.1870
	C6 Diagnostic capacity	4.2674
	C7 Blood test capacity	4.2478
	C8 Standardized treatment	4.2022

#### AHP results

The results of judgment matrix and consistency test of the indexes at each hierarchy are shown in Additional file 5. The random consistency ratio (CR) of each judgment matrix ranged from 0 to 0.0739. The combination weight of each index was calculated by the product method (Table 3). According to the results of AHP, the weights of the first-level index from high to low were infection source (0.5396), transmitting conditions (0.2970), and control capacity (0.1634). Within the infection source, the weight coefficients of the second-level index from high to low were number of imported cases (0.3382), awareness of timely medical visit of patient (0.1509), types of imported cases (0.0505). Within the transmitting conditions, the weight of *Anopheles* species (0.2475) was higher than that of *Anopheles* density (0.0495). Within the control capacity, the weight coefficients of the second-level index from high to low were prevention and control system (0.0549), work execution (0.0393), diagnostic capacity (0.0207), blood test capacity (0.0146), standardized treatment (0.0110), availability of drugs (0.0098), financial support (0.0073), and staff training (0.0058).

**Table 3:** Combination weight of the risk assessment framework

First-level index and normalized weight (w1)	Second-level index	Normalized weight (w2)	Combination weight (w1 × w2)
A Infection source 0.5396	A1 No. of imported cases	0.6267	0.3382
	A2 Types of imported cases	0.0936	0.0505
	A3 Awareness of timely medical visit of patient	0.2797	0.1509
B Transmitting conditions 0.2970	B1 Anopheles species	0.8333	0.2475
	B2 Anopheles density	0.1667	0.0495
C Control capacity 0.1634	C1 Prevention and control system	0.3357	0.0549
	C2 Financial support	0.0445	0.0073
	C3 Staff training	0.0356	0.0058
	C4 Work execution	0.2405	0.0393
	C5 Availability of drugs	0.0602	0.0098
	C6 Diagnostic capacity	0.1267	0.0207
	C7 Blood test capacity	0.0896	0.0146
	C8 Standardized treatment	0.0672	0.0110

### Comparison of the results obtained by Delphi + AHP and only Delphi method

A comparison of the results obtained by Delphi + AHP and the Delphi only method was made to determine if there was an additional benefit of AHP (Table 4). In Delphi + AHP result, the number of imported cases was more important than *Anopheles* species and types of imported cases was more important than work execution and *Anopheles* density. The rank of other indexes did not change. After AHP, the weight gaps between indexes were obviously enlarged.

**Table 4:** Comparison of the results obtained by Delphi + AHP and only Delphi method

First-level index	Second-level index	Weight (Delphi + AHP)	Weight (only Delphi)	Rank (Delphi + AHP)	Rank (only Delphi)	Rank change
A Infection source0.5396	A1 No. of imported cases	0.3382	4.5391	1	2	1
	A2 Types of imported cases	0.0505	4.3500	5	7	2
	A3 Awareness of timely medical visit of patient	0.1509	4.4217	3	3	0
B Transmitting conditions0.2970	B1 Anopheles species	0.2475	4.5804	2	1	-1
	B2 Anopheles density	0.0495	4.3565	6	6	0
C Control capacity0.1634	C1 Prevention and control system	0.0549	4.4087	4	4	0
	C2 Financial support	0.0073	4.1217	12	12	0
	C3 Staff training	0.0058	4.0065	13	13	0
	C4 Work execution	0.0393	4.3870	7	5	-2
	C5 Availability of drugs	0.0098	4.1870	11	11	0
	C6 Diagnostic capacity	0.0207	4.2674	8	8	0
	C7 Blood test capacity	0.0146	4.2478	9	9	0
	C8 Standardized treatment	0.0110	4.2022	10	10	0

## Discussion

Malaria is a disease that can rebound dramatically after periods of control. The re-introduction of malaria has been recorded in many countries and regions of the world after initial elimination [10–16]. As China and other countries approach malaria elimination goals, tools are required to evaluate the transmission risk caused by imported malaria cases to ensure that elimination is maintained and that post-elimination resources can be used effectively. There are many factors that can influence transmission and their interactions are complex. There is existing literature of the development of transmission risk index systems for imported malaria [17–20], but it can be challenging to obtain accurate quantitative risk data from field studies, especially given the low number of cases, the heterogeneity of transmission dynamics and rapid changes of these dynamics over time.

It may be possible to rapidly quantify key transmission risk indexes using expert opinion. Although the Delphi method is a well-established method for assessing expert opinion in other fields, little is known about its application to malaria and its combination with the AHP for an assessment of malaria transmission risk from imported cases [19]. A practical assessment tool that is objectively derived from expert opinion has the potential to assist greatly in simplifying the evaluation of imported cases and, accurately assessing the degree of risk.

This study used the Delphi methods and AHP to identify 13 important transmission risk factors for malaria. In particular, AHP, enabled the identification of the top three risk factors. This included the number of imported cases, *Anopheles* species, and awareness of timely medical visit of patient. This approach helped to decrease the focus towards other less important transmission risk indexes, thus potentially aiding the health system to put its resources in priority areas and take necessary action to prevent re-introduction or resurgence of malaria.

There are several approaches that can be adopted to respond to these identified risk indexes. High numbers of imported cases in a region can be used to strengthen cooperation with customs entry and exit administration, and to carry out targeted high-sensitivity detection among population arriving from epidemic areas. In areas where there are more than one main vector species, greater vigilance and enhanced vector surveillance and control can be implemented. In terms of awareness of timely medical visit of patient, it may be possible to strengthen the monitoring and analysis of the length of time from symptom onset to first medical visit. Increased efforts for health education and publicity may be needed for the target population. With this weighted index system, risk assessments can be conducted in pre-elimination or elimination areas, relevant data can be collected, and targeted testing and prevention measures can be undertaken for high-risk regions.

When comparing the combined Delphi and AHP method with the Delphi method only, the ranking of second-level indexes did not change much, but the AHP did assist in quantifying the relative importance of risk indexes. Firstly, this helped in the selection of assessment indexes by ensuring the quality of evaluation while optimizing and simplifying the evaluation of the different possible indexes. This greatly aids the operability and practicability of a subsequent risk assessment. A more rapid assessment could be implemented by using the top five or ten indexes in regions where the data is difficult to collect or if the assessment needs to be conducted quickly. Secondly, constructing the judgment matrix according to the importance score of the Delphi method can avoid the influence of subjective factors to a greater extent and make the results more objective and credible. Thirdly, by combining AHP, the weight gap between indexes could be enlarged, which is conducive to further discovering the key risk indexes and making the identification of important risk factors more refined. Fourthly, the AHP was conducted after the Delphi method had screened out the main risk indexes from a wide range of other potential risk factors. This sequential methodology thus reduced the complexity of the statistical calculations that might have been required because of excessive variables. The calculation method is therefore relatively simple and does not require particularly complex software.

There are some other studies that have focused on establishing malaria risk assessment systems or models [19, 21, 22]. Compared with these studies, our approach did not include meteorological or socio-economic indicators. It was considered that terrain (historical environment), annual average temperature, annual rainfall, and local economic levels were indirect factors influencing the vector or human-mosquito contact, not the core factors directly influence the transmission of malaria [23] and the weight was not heavy. Studies that include these indicators tend to predict the occurrence of the disease and calculate absolute risk especially in area with more imported cases and relatively high risk. Such studies tend to be more precise and strict about the time points of assessment. Our model could be more inclined to assess relative risks between different regions in areas with less imported cases and relatively low risk. Our approach sought to pay more attention to vital advice for key measures.

## Conclusions

This study has established a system to evaluate local transmission risk from imported cases using a group of quantified risk indexes using the Delphi method and AHP. In this risk assessment framework, 13 indexes at 3 levels are selected and used to evaluate the risk situation of local transmission based on infection source, transmission conditions, and surveillance and response capacity. This provides a practical, and objective tool to guide risk management and response and to support malaria elimination activities as an important public health strategy. It is recommended to collect the data in recent several years and calculate the comprehensive scores so as to identify the current risk factors and to implement corresponding interventions.

It is anticipated that this transmission risk assessment index system will not be fixed but will need to be constantly improved in its application according to the local situation and practice. By establishing a system of risk assessment, we aim to continually evaluate and refine risk factors, focus monitoring activities, and implement prevention activities in areas of greatest need to sustain malaria elimination.

## Additional files

Additional file 1: Multilingual abstracts in the five official working languages of the United Nations (pdf 376 kb)

Additional file 2: Information of the Delphi experts (DOCX 16 kb)

Additional file 3: The value tables (DOCX 14 kb)

Additional file 4: The indexes for the two rounds of the Delphi process (DOCX 15 kb)

Additional file 5: Judgment matrix of the indexes (DOCX 17 kb)

## Data Availability

Please contact author for data requests.

## Abbreviations

AHP: Analytic Hierarchy Process
Ca: Expert’s judgment criteria coefficient
CI: Consistency index
CR: Average random consistency index
Cr: Expert’s authority coefficient
Cs: Expert’s familiarity coefficient
WHO: World Health Organization
