# Supplementary material for: A framework for assessing local transmission risk of imported malaria cases
Source: Infect Dis Poverty. 2019 Jun 7;8:43. doi: 10.1186/s40249-019-0552-6 (PMC6555958; doi:10.1186/s40249-019-0552-6)
Supplement: Supplementary file 1 — Multilingual abstracts in the five official working languages of the United Nations (pdf 376 kb) [file 40249_2019_552_MOESM1_ESM.pdf]

Translation of the abstract into the five official working languages of the United Nations

المستوردة الملاريا لحالات المحلية الانتقال مخاطر لتقييم اطار

شياو ونينغ تشانغ زاي شاو ، غونغ فنغ يان ، لي هونغ تشي ، ريتشاردز .س جاك ، لي لي

مجرده
عن الناجم المحلي الانتقال منع إلى الحاجة الملاريا علي القضاء وادامه تحقيق في الرئيسية المسائل من الخلفية: استخدامها ويمكن ، المحلية العوامل من مجموعه علي ذلك حدوث احتمال ويعتمد .المستوردة الملاريا حالات المخاطر مؤشرات لتحديد وأدارتها المخاطر لتقييم استراتيجية وضع يلزم ، ولذلك .الإرسال لاحتواء موارد لتخصيص لإعطاء بينها والجمع كميا المخاطر هذه تحديد أيضا ويلزم .مستورده حالات حدوث عند الملاريا بانتقال المتعلقة وفقا العدوى انتقال لمنع اداريه منطقه لكل الموارد لتخصيص ذلك استخدام ويمكن .المرجحة المخاطر مؤشر درجه المخاطرة لدرجه.
الأساليب: تم توليد قائمه بمؤشرات المخاطر المحتملة من استعراض الأدبيات ومشاورات الخبراء وحلقات النقاش. وظروف العدوى مصدر ذلك في بما الأول المستوي من فهارس 4 في البداية في المؤشرات هذه صنفت وقد ثم .تفصيلا أكثر الثاني المستوي فهارس إلى هذه من كل توسيع تم ثم .التحكم وقدره السكان وضعف الإرسال متعاقبتين جولتين خلال هذه المخاطر مؤشرات وتنقيح لاستعراض الخبراء راي علي للحصول دلفي طريقه استخدمت لمخاطر النهائي الإطار في المخاطر مؤشرات وأدرجت .أهميتهم مستوي بشان الخبراء بين فيما الاتفاق حجم لتحديد الوزن لحساب التحليلي الهرمي التسلسل عمليه استخدمت المرجحة ≤ 4. ثم الاهميه درجه حققت إذا الإرسال مخاطر لتقييم استخدامه يمكن تقييم اطار لإنشاء ذلك استخدم ثم .النهائية المخاطر فهارس من لكل المخصص المجالات مختلف في المحلية الإرسال.
النتائج: أجريت جولتان من المشاورات في دلفي. واستخدم ثلاثه وعشرون خبيرا في كل جولة بمعدل استرداد 100 في المائة من استبيانات المشاركين. وكانت معاملات التنسيق (W) لجولتي التشاور دلفي 0.341 و 0.423 ، علي التوالي (P < 0.05). وتم تحديد ثلاثه فهارس من المستوي الأول و 13 مؤشرا من المستوي الثاني. تم تنفيذ عمليه التسلسل الهرمي التحليلي لحساب وزن الفهارس .النسبة لفهارس المستوي الأول ، ومصدر العدوى ، وظروف الإرسال ، وقدره التحكم ، كان وزن المؤشر 0.5396 ، 0.2970 و 0.1634 علي التوالي. النسبة للمؤشرات الثلاثة العليا من المستوي الثاني ، وعدد حالات الملاريا المستوردة ، وأنواع انوهلليس ، والوعي بالزيارة الطبية للمريض في الوقت المناسب ، كان وزن المؤشر 0.3382 ، 0.2475 ، و 0.1509 علي التوالي.
الاستنتاجات: تم إنشاء نظام مفهرس لتقييم مخاطر الإرسال بالنسبة للملاريا المستوردة باستخدام طريقه دلفي وعمليه التسلسل الهرمي التحليلي. واعتبر ان هذا الأمر يشكل أداه موضوعيه وعمليه لتقييم المخاطر التي تتعرض لها الشركة من حالات الملاريا المستوردة إلى الصين.

Translated from English version into Arabic by Hamza Shaffison

输入性疟疾病例当地传播风险的评估体系

Lei Lei, Jack S. Richards, Zhi-Hong Li, Yan-Feng Gong, Shao-Zai Zhang and Ning Xiao

目的：为了达到消除疟疾目标和维持消除疟疾状态，当前面临的一项重要议题是如何防止输入性疟疾引起本地传播。这主要取决于一系列当地影响疾病传播和流行的因素，通过研究这些风险因素可为采取有针对性的措施来限制或阻断传播提供科学依据。本研究的主要目的是建立输入性疟疾引起继发传播的风险评估指标体系，为科学客观的评价输入性疟疾的传播风险评估提供基础。

方法：在文献复习、专家咨询和小组讨论的基础上，筛选出拟用于输入性疟疾传播风险评估的各项一级指标和二级指标，然后分别采用德尔菲法筛选出主要评估指标，采用层次分析法确定主要评估指标的权重，建立输入性疟疾传播风险评价指标体系。
结果：全程共进行了2轮德尔菲专家咨询，第1轮和第2轮分别发送咨询问卷23份，全部回收，回收率100%，2轮德尔菲咨询的专家协调系数（*W*）分别为0.341、0.423（*P*<0.05），经德尔菲法筛选出一级评估指标3个，二级评估指标13个。采用层次分析法得到各项评估指标权重，其中一级指标“传染源”权重为0.5396，“传播条件”权重为0.2970，“防控能力”权重为0.1634，二级指标权重排名前三的分别为“输入性病例数量”0.3382，“媒介种群”0.2475，“病例及时就诊意识”0.1509。
结论：应用德尔菲法和层次分析法构建的输入性疟疾传播风险评估指标体系具有的一定的科学性、客观性和可利用性，为输入性疟疾风险评估提供了基础，为明确防控重点提供了科学依据。

Translated from English version into Chinese by Lei Lei

Cadre d’évaluation du risque de transmission locale du paludisme importé

Lei Lei, Jack S. Richards, Zhi-Hong Li, Yan-Feng Gong, Shao-Zai Zhang et NingXiao

Résumé
Contexte : Dans l’optique de l’élimination durable du paludisme, il est nécessaire d’empêcher la transmission locale du parasite à partir de cas importés. La probabilité de cette transmission dépend de nombreux facteurs locaux et peut être utilisée pour attribuer les ressources nécessaires afin de contenir la transmission. Une stratégie d’évaluation et de gestion des risques est donc nécessaire pour identifier les index de risque de transmission de la maladie en présence de cas importés. Ces risques doivent également être quantifiés et combinés afin d’obtenir un score d’index de risque pondéré, qui pourra être utilisé pour affecter les ressources à chaque région administrative, selon son degré de risque, afin d’empêcher la transmission.
Méthodes : Une liste des index de risque potentiels a été générée à partir d’une revue de la littérature, de la consultation d’experts et de discussions en groupe de travail. Nous avons identifié, dans un premier temps, quatre index primaires : source de l’infection, conditions de la transmission, vulnérabilité de la population et capacité de lutte. Chacun a ensuite été détaillé en index secondaires. La méthode de Delphes a été utilisée pour solliciter l’avis d’experts sur ces index de risque en deux tours consécutifs, afin de quantifier le consensus entre les experts sur leur niveau d'importance. Les index de risque ont été inclus dans le cadre de risque de transmission final s’ils atteignaient un score d’importance pondérée égal ou supérieur à 4. Le processus de hiérarchie analytique multicritère a ensuite été utilisé pour calculer le poids de chacun des index de risque finaux, puis utilisé pour créer un cadre d’évaluation qui peut être utilisé pour évaluer le risque de transmission local dans différentes zones.
Résultats : Deux tours de consultation selon la méthode de Delphes ont été menés. Vingt-trois experts ont été consultés à chaque tour et le taux de retour des questionnaires était de 100 %. Les coefficients de coordination (*W*) pour les deux tours de consultation étaient respectivement de 0,341 et 0,423 (*P* < 0,05). Trois index primaires et treize index secondaires ont été identifiés. Le processus

d’analyse hiérarchique multicritère a été exécuté pour calculer le poids des index de risque. La pondération des index primaires était de 0,5396 pour la source d’infection, 0,2970 pour les conditions de transmission et 0,1634 pour la capacité de lutte. Pour les trois principaux index secondaires, la pondération était de 0,3382 pour le nombre de cas de paludisme importés, 0,2475 pour l’espèce d’ *Anopheles* et 0,1509 pour la connaissance par le patient de la nécessité d’une visite médicale rapide.

Conclusions : Un système indexé d’évaluation du risque de transmission du paludisme importé a été créé à l’aide de la méthode de Delphes et du processus d’analyse hiérarchique multicritère. Il a été jugé objectif et pratique pour l’évaluation du risque de transmission de cas de paludisme importés en Chine.

Translated from English version into French by Suzanne Assenat, Revised by Iris Soliman, through

TRANSLATORS WITHOUT BORDERS

Система оценки риска регионального распространения случаев завозной малярии

Лей Лей, Джэк С. Ричардс, Жи-Хонг Ли, Ян-Фенг Гонг, Шао-Зай Жанг и Нинг Ксао

Резюме

Обоснование: Ключевой проблемой ликвидации малярии является профилактика распространения завозных случаев инфекции. Вероятность такого распространения зависит от ряда факторов, характерных для каждого региона, которые могут быть использованы для распределения ресурсов для сдерживания распространения инфекции. Следовательно, для определения показателей значимости рисков распространения малярии при завозных случаях необходима стратегия оценки рисков и управления ими. Эти риски также должны быть определены количественно и объединены для получения взвешенной оценки коэффициента риска распространения инфекции. Данный показатель впоследствии может быть использован при распределении ресурсов для профилактики распространения инфекции для каждого административного округа в соответствии со степенью риска.

Методы: На основе обзора литературы, консультаций со специалистами и группового обсуждения был составлен список коэффициентов потенциальных рисков. Первоначально было выделено 4 коэффициента первого уровня, включая источник инфекции, пути передачи, восприимчивость населения и возможности контроля распространения. Затем каждый из данных коэффициентов был подразделен на более специфичные коэффициенты второго уровня. Для получения экспертного заключения по анализу и проверке коэффициентов риска в течение двух последовательных раундов использовался метод Дельфи для количественного определения согласия между экспертами в отношении уровня значимости данных коэффициентов. Коэффициенты риска включались в окончательную структуру риска распространения инфекции, если они достигали взвешенной оценки значимости ≥ 4. Методом анализа иерархий было вычислено значение каждого из окончательных

коэффициентов риска. Эти данные были использованы для создания системы оценки, которая может использоваться для определения степени риска локального распространения инфекций в различных регионах.

Результаты: Было проведено два раунда консультаций методом экспертных оценок. В каждом раунде было задействовано 23 эксперта, возврат опросных листов от участников составил 100 %. Коэффициент согласия экспертов в двух раундах консультаций методом экспертных оценок (W) составил 0,341 и 0,423 соответственно ($P < 0{,}05$). Были определены три коэффициента первого уровня и 13 — второго уровня. Методом анализа иерархий была вычислена значимость каждого коэффициента. Коэффициенты первого уровня — источник инфекции, пути передачи и степень контроля — получили значения 0,5396, 0,2970 и 0,1634 соответственно. Первые три коэффициента второго уровня — количество случаев завозной малярии, вид анофелесов и осведомленность пациента о необходимости своевременного обращения за медицинской помощью — получили значения 0,3382, 0,2475 и 0,1509 соответственно.

Заключение: С помощью метода экспертных оценок и метода анализа иерархий была разработана система коэффициентов для оценки риска распространения завозной малярии. Данная система была признана практическим инструментом для объективной оценки риска распространения завозной малярии в Китае.

Translated from English version into Russian by by Nataliya Zhydkikh, Revised by Michael Orlov, through

Un marco para evaluar el riesgo de transmisión local de los casos de malaria importada

Lei Lei, Jack S. Richards, Zhi-Hong Li, Yan-Feng Gong, Shao-Zai Zhang y Ning Xiao

Resumen

Antecedentes: una cuestión clave para lograr la eliminación de la malaria y mantenerla es la necesidad de prevenir la transmisión local derivada de casos de malaria importada. La probabilidad de que esto ocurra depende de una variedad de factores locales, que pueden usarse para asignar recursos a fin de contener la transmisión. Por lo tanto, se requiere una estrategia de evaluación y gestión del riesgo para identificar los índices de riesgo de la trasmisión cuando ocurren casos importados. Estos riesgos también deben cuantificarse y combinarse para obtener un puntaje del índice de riesgo ponderado. Este puede utilizarse para asignar los recursos a cada región administrativa con el fin de evitar la transmisión de acuerdo con el grado de riesgo.

Métodos: a partir de una revisión bibliográfica, una consulta de expertos y un panel de discusión se generó una lista de índices de riesgos potenciales. Se clasificaron inicialmente en cuatro índices de primer nivel, incluidas la fuente de infección, las condiciones de trasmisión, la vulnerabilidad poblacional y la capacidad de control. Después, cada uno de estos se amplió a índices de segundo

nivel más detallados. Luego se usó el método Delphi para obtener la opinión de expertos a fin de examinar y revisar estos índices de riesgo en dos rondas consecutivas con el propósito de cuantificar el acuerdo entre los expertos en cuanto a su nivel de importancia. Si los índices de riesgo alcanzaron un puntaje de importancia ponderada ≥ 4, se incluyeron en el Marco de Riesgo de Transmisión final. El Proceso de Jerarquía Analítica se utilizó para calcular la importancia asignada a cada uno de los índices de riesgo finales. Esto se usó después para crear un marco de evaluación que se puede usar para evaluar el riesgo de transmisión local en diferentes áreas.

Resultados: se llevaron a cabo dos rondas de consulta Delphi. Veintitres peritos se utilizaron en cada ronda con una tasa de recuperación del 100 % de los cuestionarios de los participantes. Los coeficientes de coordinación (*W*) para las dos rondas de la consulta Delphi fueron 0,341 y 0,423, respectivamente ($P < 0{,}05$). Se identificaron tres índices de primer nivel y 13 de segundo nivel. El Proceso de Jerarquía Analítica se utilizó para calcular el peso de los índices. Para los índices de primer nivel, la fuente de infección, las condiciones de trasmisión y la capacidad de control, el peso de cada índice fue de 0,5396, 0,2970 y 0,1634, respectivamente. Para los tres índices principales de segundo nivel, el número de casos de malaria importada, la *especie Anopheles* y el conocimiento del paciente sobre la visita médica oportuna, el peso de cada índice fue de 0,3382, 0,2475 y 0,1509, respectivamente.

Conclusiones: se estableció un sistema indexado de evaluación del riesgo de transmisión para la malaria importada utilizando el método Delphi y el Proceso de Jerarquía Analítica. Esto se valoró como una herramienta objetiva y práctica para la evaluación del riesgo de transmisión de la malaria producida por casos importados a China.

Translated from English version into Spanish by Patricia Cassoni, Revised by Mayra León, through

TRANSLATORS
WITHOUT BORDERS
