# Supplementary material for: A framework for assessing local transmission risk of imported malaria cases
Source: Infect Dis Poverty. 2019 Jun 7;8:43. doi: 10.1186/s40249-019-0552-6 (PMC6555958; doi:10.1186/s40249-019-0552-6)
Supplement: Supplementary file 2 — Information of the Delphi experts (DOCX 16 kb) [file 40249_2019_552_MOESM2_ESM.docx]

Additional file 2: Information of the Delphi experts

| No. expert | Gender | Age | Institution | Title | Field of expertise |
| --- | --- | --- | --- | --- | --- |
| 1 | Male | 53 | National Institute of Parasitic Diseases, Chinese Center for Disease Control and Prevention, Shanghai, China. | Professor | Pathogen biology and control technology of parasitic diseases |
| 2 | Male | 48 | National Institute of Parasitic Diseases, Chinese Center for Disease Control and Prevention, Shanghai, China. | Professor | Malaria control |
| 3 | Male | 40 | National Institute of Parasitic Diseases, Chinese Center for Disease Control and Prevention, Shanghai, China. | Professor | Malaria control |
| 4 | Male | 42 | National Institute of Parasitic Diseases, Chinese Center for Disease Control and Prevention, Shanghai, China. | Professor | Malaria control |
| 5 | Male | 47 | National Institute of Parasitic Diseases, Chinese Center for Disease Control and Prevention, Shanghai, China. | Professor | Epidemiology and parasitic diseases control |
| 6 | Male | 39 | Jiangsu Institute of Parasitic Diseases, Wuxi, China. | Professor | Malaria control |
| 7 | Male | 60 | Yunnan Institute of Parasitic Diseases, Puer, China. | Professor | Malaria control |
| 8 | Male | 42 | Yunnan Institute of Parasitic Diseases, Puer, China. | Professor | Malaria control |
| 9 | Male | 52 | Hubei Provincial Center for Disease Control and Prevention, Wuhan, China | Professor | Parasitic diseases prevention and control |
| 10 | Male | 48 | Hebei Provincial Center for Disease Control and Prevention, Shijiazhuang, China | Professor | Epidemiology |
| 11 | Male | 53 | Zhejiang Provincial Center for Disease Control and Prevention, Hangzhou, China | Professor | Parasitic diseases and malaria control |
| 12 | Male | 48 | Anhui Provincial Center for Disease Control and Prevention, Hefei, China | Associate professor | Endemic diseases and malaria control |
| 13 | Female | 59 | Jiangxi Provincial Center for Disease Control and Prevention, Nanchang, China | Professor | Vector control |
| 14 | Male | 57 | Jiangxi Provincial Center for Disease Control and Prevention, Nanchang, China | Associate professor | Endemic diseases and parasitic diseases control |
| 15 | Male | 59 | Jiangxi Provincial Center for Disease Control and Prevention, Nanchang, China | Professor | Malaria control |
| 16 | Female | 52 | Jiangxi Provincial Center for Disease Control and Prevention, Nanchang, China | Associate professor | parasite control |
| 17 | Male | 51 | Jiangxi Provincial Center for Disease Control and Prevention, Nanchang, China | Associate professor | Mosquito ecology and mosquito-borne infectious diseases |
| 18 | Female | 44 | Nanchang Center for Disease Control and Prevention, Nanchang, China | Associate professor | Endemic diseases and parasitic diseases control |
| 19 | Female | 49 | Pingxiang Center for Disease Control and Prevention, Pingxiang, China | Associate professor | Parasite control |
| 20 | Male | 40 | Ji'an Center for Disease Control and Prevention, Ji'an, China | Associate professor | Endemic diseases and malaria control |
| 21 | Female | 49 | Qingshanhu Center for Disease Control and Prevention, Nanchang, China | Professor | Infectious diseases prevention and control |
| 22 | Male | 43 | Pengzhe Center for Disease Control and Prevention, Jiujang, China | Associate professor | Infectious diseases prevention and control |
| 23 | Male | 50 | Wangnian Center for Disease Control and Prevention, Shangrao, China | Associate professor | Infectious diseases prevention and control |
