# Supplementary material for: A framework for assessing local transmission risk of imported malaria cases
Source: Infect Dis Poverty. 2019 Jun 7;8:43. doi: 10.1186/s40249-019-0552-6 (PMC6555958; doi:10.1186/s40249-019-0552-6)
Supplement: Supplementary file 3 — The value tables (DOCX 14 kb) [file 40249_2019_552_MOESM3_ESM.docx]

Additional file 3: The value tables

| Table 1 Level of importance of the indexes | |
| --- | --- |
| Level of importance | Score |
| Very important | 5 |
| Important | 4 |
| Moderately important | 3 |
| Unimportant | 2 |
| Most unimportant | 1 |

| Table 2 Expert’s judgment criteria for the indexes (Ca) | | | |
| --- | --- | --- | --- |
| Basis of judgment | The degree of influence on expert judgment | | |
|  | Great | General | Little |
| Theoretical analysis | 0.3 | 0.2 | 0.1 |
| Practical experience | 0.5 | 0.4 | 0.3 |
| Learn from your peers | 0.1 | 0.1 | 0.1 |
| Intuitive feeling | 0.1 | 0.1 | 0.1 |

| Table 3 Expert’s familiarity with the indexes (Cs) | |
| --- | --- |
| Familiarity scale | Cs |
| Very familiar | 1.0 |
| Familiar | 0.8 |
| Moderately familiar | 0.5 |
| Unfamiliar | 0.2 |
| Most unfamiliar | 0 |
