# Supplementary material for: A framework for assessing local transmission risk of imported malaria cases
Source: Infect Dis Poverty. 2019 Jun 7;8:43. doi: 10.1186/s40249-019-0552-6 (PMC6555958; doi:10.1186/s40249-019-0552-6)
Supplement: Supplementary file 4 — The indexes for the two rounds of the Delphi process (DOCX 15 kb) [file 40249_2019_552_MOESM4_ESM.docx]

Additional file 4: The indexes for the two rounds of the Delphi process

| The indexes for the first round of Delphi | The indexes for the second round of Delphi |
| --- | --- |
| *First-level indexes*  1 Infection source | 1 Infection source |
| 2 Transmitting conditions | 2 Transmitting conditions |
| 3 Population vulnerability | 3 Population vulnerability |
| 4 Control capacity | 4 Control capacity |
| *Second-level indexes*  1.1 Population inflow from the epidemic area | 1.1 Population inflow from the epidemic area recently |
| 1.2 Number of imported cases | 1.2 Number of imported cases recently |
| 1.3 Types of imported cases | 1.3 Types of imported cases recently |
| 1.4 Health seeking behaviour | 1.4 Awareness of timely medical visit of patient |
|  | 1.5 Inform about the travel history |
|  | 1.6 Liquidity of people in incubation period |
| 2.1 Temperature | 2.1 Temperature |
| 2.2 Relative humidity | 2.2 Relative humidity |
| 2.3 Rainfall | 2.3 Rainfall |
| 2.4 Terrain | 2.4 Terrain |
| 2.5 Crop planting, vegetation | 2.5 Crop planting, vegetation |
| 2.6 *Anopheles* species | 2.6 *Anopheles* species |
| 2.7 *Anopheles* density | 2.7 *Anopheles* density |
| 2.8 Sensitivity of the insecticide | 2.8 Sensitivity of the insecticide |
| 2.9 Historical prevalence of malaria | 2.9 Historical prevalence of malaria |
|  | 2.10 Data of large livestock (pigs, cows, etc.) |
|  | 2.11 Varieties and quantities of insecticide |
|  | 2.12 Anopheles transmission energy (blood-sucking habit, biting rate, etc.) |
| 3.1 Population antibody level | 3.1 Population antibody level |
| 3.2 Population knowledge of malaria | 3.2 Population knowledge of malaria |
| 3.3 Local economic level | 3.3 Local economic level |
| 3.4 Living conditions and protective habits | 3.4 Living conditions and protective habits |
| 3.5 Working environment | 3.5 Working environment and working hours |
| 3.6 Population mobility (including aggregation) | 3.6 Population mobility (including aggregation) |
|  | 3.7 Drug resistance |
| 4.1 Organization and management | 4.1 Organization and management |
| 4.2 Prevention and control system | 4.2 Prevention and control system |
| 4.3 Financial support | 4.3 Financial support |
| 4.4 Supervise the quality control | 4.4 Supervise the quality control |
| 4.5 Health promotion | 4.5 Health promotion |
| 4.6 Staff training | 4.6 Staff training |
| 4.7 Monitoring and evaluation | 4.7 Monitoring and evaluation |
| 4.8 Work execution | 4.8 Work execution |
| 4.9 Availability of drugs | 4.9 Availability of drugs |
|  | 4.10 Awareness among medical staff |
|  | 4.11 Diagnostic capacity |
|  | 4.12 Blood test capacity |
|  | 4.13 Highly sensitive screening tools |
|  | 4.14 Standardized treatment |
