# Supplementary material for: A framework for assessing local transmission risk of imported malaria cases
Source: Infect Dis Poverty. 2019 Jun 7;8:43. doi: 10.1186/s40249-019-0552-6 (PMC6555958; doi:10.1186/s40249-019-0552-6)
Supplement: Supplementary file 5 — Judgment matrix of the indexes (DOCX 17 kb) [file 40249_2019_552_MOESM5_ESM.docx]

Additional file 5: Judgment matrix of the indexes

Table1 Judgment matrix of first-level indexes

| First-level index | Infection source | Transmitting conditions | Control capacity | Initial weight  w' | Normalized weight  w |
| --- | --- | --- | --- | --- | --- |
| Infection source | 1 | 2 | 3 | 1.8171 | 0.5396 |
| Transmitting conditions | 1/2 | 1 | 2 | 1.0000 | 0.2970 |
| Control capacity | 1/3 | 1/2 | 1 | 0.5503 | 0.1634 |

Note: λmax=3.0092 ，CI=0.0046 ，CR=0.0079

Table2 Judgment matrix of second-level indexes relating to infection source (1)

| Second-level index | No. of imported cases | Types of imported cases | Awareness of timely medical visit of patient | Initial weight  w' | Normalized weight  w |
| --- | --- | --- | --- | --- | --- |
| No. of imported cases | 1 | 5 | 3 | 2.4662 | 0.6267 |
| Types of imported cases | 1/5 | 1 | 1/4 | 0.3684 | 0.0936 |
| Awareness of timely medical visit of patient | 1/3 | 4 | 1 | 1.1006 | 0.2797 |

Note: λmax=3.0858 ，CI=0.0429 ，CR=0.0739

Table3 Judgment matrix of second-level indexes relating to transmitting conditions (2)

| Second-level index | Anopheles species | Anopheles density | Initial weight  w' | Normalized weight  w |
| --- | --- | --- | --- | --- |
| Anopheles species | 1 | 5 | 2.2361 | 0.8333 |
| Anopheles density | 1/5 | 1 | 0.4472 | 0.1667 |

Note: λmax=2 ，CI=0 ，CR=0

Table4 Judgment matrix of second-level indexes relating to control capacity (3)

| Second-level index | Prevention and control system | Financial support | Staff training | Work execution | Availability of drugs | Diagnostic capacity | Blood test capacity | Standardized treatment | Initial weight  w' | Normalized weight  w |
| --- | --- | --- | --- | --- | --- | --- | --- | --- | --- | --- |
| Prevention and control system | 1 | 6 | 6 | 2 | 6 | 3 | 3 | 5 | 3.4363 | 0.3357 |
| Financial support | 1/6 | 1 | 2 | 1/5 | 1/2 | 1/3 | 1/3 | 1/2 | 0.4555 | 0.0445 |
| Staff training | 1/6 | 1/2 | 1 | 1/5 | 1/2 | 1/3 | 1/3 | 1/3 | 0.3641 | 0.0356 |
| Work execution | 1/2 | 5 | 5 | 1 | 4 | 3 | 3 | 3 | 2.4620 | 0.2405 |
| Availability of drugs | 1/6 | 2 | 2 | 1/4 | 1 | 1/2 | 1/2 | 1/2 | 0.6164 | 0.0602 |
| Diagnostic capacity | 1/3 | 3 | 3 | 1/3 | 2 | 1 | 2 | 2 | 1.2968 | 0.1267 |
| Blood test capacity | 1/3 | 3 | 3 | 1/3 | 1/2 | 1/2 | 1 | 2 | 0.9170 | 0.0896 |
| Standardized treatment | 1/5 | 2 | 3 | 1/3 | 1/2 | 1/2 | 1/2 | 1 | 0.6877 | 0.0672 |

Note: λmax=8.0157 ，CI=0.0026，CR=0.0019
